# Battle in the New World: *Helicoverpa armigera* versus *Helicoverpa zea* (Lepidoptera: Noctuidae)

**DOI:** 10.1371/journal.pone.0167182

**Published:** 2016-12-01

**Authors:** José P. F. Bentivenha, Silvana V. Paula-Moraes, Edson L. L. Baldin, Alexandre Specht, Ivana F. da Silva, Thomas E. Hunt

**Affiliations:** 1 Department of Entomology and Acarology—Luiz de Queiroz College of Agriculture, University of Sao Paulo, Piracicaba, São Paulo, Brazil; 2 Entomology and Nematology Department, WFREC, University of Florida, Jay, Florida, United States of America; 3 Department of Crop Protection, College of Agronomic Sciences, São Paulo State University, Botucatu, São Paulo, Brazil; 4 Embrapa Cerrados, Planaltina, Distrito Federal, Brazil; 5 Haskell Agricultural Laboratory, University of Nebraska, Concord, Nebraska, United States of America; Pennsylvania State University, UNITED STATES

## Abstract

The corn earworm *Helicoverpa zea* (Boddie) and the old world bollworm *Helicoverpa armigera* (Hübner) (Lepidoptera: Noctuidae) are allopatric species and occur in important agricultural crops. In maize, both species tend to infest the ear. The introduction of *H*. *armigera* in Brazil has created a new scenario, where these *Helicoverpa* species might cohabit and interact with one another, affecting the prevalence of each species in the agroecosystem, integrated pest management, and insect resistance management. In this study, larval occurrence and proportion of these species in maize was assessed in three regions of Brazil during three crop seasons. Interaction between the species was evaluated in interspecific and intraspecific scenarios under laboratory and field conditions. *Helicoverpa zea* was predominant in Rio Grande do Sul and the Planaltina, DF (central Brazil). In western Bahia, *H*. *zea* was predominant in the first collection, but approximately equal in number to *H armigera* in the second crop season. Both species exhibit high cannibalism/predation rates, and larval size was the primary factor for larval survival in the interaction studies. Larva of *H*. *zea* had higher survival when interacting with *H*. *armigera*, indicating that *H*. *zea* has an advantage in intraguild interactions with *H*. *armigera* in maize. Overall, the results from this study indicate that maize might play a role as a source of infestation or a sink of insecticide or Bt protein unselected *H*. *armigera* populations, depending on the *H*. *zea*:*H*. *armigera* intraguild competition and adult movement in the landscape.

## Introduction

The Heliothinae (Lepidoptera: Noctuidae) is a cosmopolitan subfamily of noctuid moths that comprises more than 400 species [1]. A review involving the corn earworm complex proposed the genera *Helicoverpa* [2]. One of the significant contributions of this review was to propose the species differentiation between *Helicoverpa zea* (Boddie) and *Helicoverpa armigera* (Hübner) based on male and female genitalia.

*Helicoverpa zea* and *H*. *armigera* are allopatric species that have a preference to feed on plant reproductive tissues, often causing economic damage [3]. *Helicoverpa zea* is only present in the American continents, and so is named the new old world bollworm. *Helicoverpa armigera*, the old world bollworm, was reported present in Asia, Africa, Europe and Australia. However, a new scenario of *H*. *zea* and *H*. *armigera* cohabiting in an American continent was reported with the detection of *H*. *armigera* in Brazil during the crop season of 2012/2013 [4–6]. Indeed, *H*. *armigera* was present in Brazil at least since 2008 [7], and the potential for it to spread throughout North and South America [8] has been confirmed by reports of its occurrence in other countries, such as Argentina [9], and in the U.S on 17 June 2015, where three *H*. *armigera* moths were collected in Florida [10].

Several concerns have been raised about the impact of the interaction between these two former allopatric species, with relevance to integrated pest management and insect resistance management. Maize is the major host of *H*. *zea*, and moths usually oviposit on silks, where the neonates feed, and then move to kernels after entering the tip of the husk. When larvae reach the third instar, they become cannibalistic and frequently only one larva survives per maize ear [11–14]. This species presents management challenges because of its biological and behavioral characteristics and rapid adaptation to chemical control (e.g. resistance to organophosphate and pyrethroid insecticides) [15–17]. One alternative to insecticide control has been the use of transgenic hybrids expressing active proteins of the bacterium *Bacillus thuringiensis* (Bt). However, cases of resistance of *H*. *zea* have already been reported on Bt maize expressing the proteins Cry1Ab [18]. *Helicoverpa armigera* is a polyphagous pest and prefers cotton and sorghum [11, 19, 20]. However, injury in maize is reported, and similarly to *H*. *zea*, *H*. *armigera* moths tend to oviposit on maize silks and larvae feed on kernels [19]. Considering this preference for reproductive tissues (silks and early ear development stages), maize supports one generation of *H*. *armigera*, and economic impact has been reported [21]. In addition, insecticide resistance of *H*. *armigera* has been reported to organophosphate, carbamate, and pyrethroid insecticides [22, 23].

In Brazil, besides maize, there is the possibility that these two species could cohabit in other economically important crops such as soybean, cotton, tomatoes, dry beans, among others [5, 6, 24]. However, maize represents a model crop to study the interaction between these two pests. The maize ear is the same feeding site for both species, where larvae are naturally confined [14, 19, 25] with less possibility of movement to other reproductive tissues on the same plant. Larval movement to other feeding sites is more likely in cotton [19] or soybean [26]. Thus, maize represents an appropriate arena for intraguild interaction studies. In addition, larvae of both species have cannibalistic behavior [12, 27, 28], which might intensify the intraguild competition.

Here we documented the larval occurrence and proportion of *H*. *zea* and *H*. *armigera* in maize ears, in three regions of Brazil, using data collected from the 2012/2013 and 2014/2015 crop seasons. The interaction between these former allopatric species was also investigated based on different interspecific and intraspecific scenarios, which were conducted under laboratory and field conditions.

## Materials and Methods

### Larval occurrence and percentage of *H*. *zea* and *H*. *armigera* in maize fields in Brazil

Maize fields were sampled in three states of Brazil (Bahia, Distrito Federal, Rio Grande do Sul) to document the occurrence and proportion of *H*. *zea* and *H*. *armigera* larvae in maize ears. Commercial fields of maize were selected based on their representative size for each region and when maize kernels were in the milk to dough stages (R3-R4) [29]. The field collections were authorized by Instituto Chico Mendes de Conservação da Biodiversidade, under SISBIO licenses n.38520-1, n.38520-2, n.38520-3, n.38520-4, and n.48218-1. In western Bahia, in the municipality of São Desidério (12°21’48”S and 44°58’24”W), larval sampling was conducted in the 2012/2013 and 2014/2015 crop seasons. In the crop season of 2012/2013, maize ears were sampled in Bt maize (Cry1Ab+Cry1F) and non-Bt maize. Each maize field was 200 hectares and approximately 300 plants were randomly inspected per field during maize stage R3 [29]. *Helicoverpa* sp. larvae at different instars were collected from maize ears, placed in 30 ml containers with artificial diet [30] and transported to the laboratory (25 ± 2°C, RH: 60 ± 10%; 14:10 [L:D]) at Embrapa Cerrados, Planaltina, DF. The larvae were held under laboratory conditions until adult emergence. In the crop season 2014/2015, *Helicoverpa* larvae were likewise collected and held under the same laboratory conditions until adult emergence.

In the experimental area of Embrapa Cerrados, municipality of Planaltina, DF (15°37’09”S and 47°39’09”W) in central Brazil, larval sampling was conducted in two non-Bt maize fields (5 hectares each field) during the 2014/2015 crop season. One field was sampled in November 2014 and approximately 80 larvae were collected. In April 2015, the other field was sampled with approximately 60 larvae collected. At both sampling dates, the maize fields were in stage R3-R4.

In the municipality of São Pedro da Serra (29°25’16”S and 51°30’48”W), Rio Grande do Sul, southern Brazil, larval sampling was conducted in two maize fields during the 2015/2016 crop season. Both fields were non-Bt maize and 5 hectares each. The collected larvae were transported to Embrapa Cerrados, Planaltina, DF and held under the same laboratory conditions previously described.

Insects that reached the adult stage in each collection were identified by genitalia dissection [31], and identification based on morphological characters described in the literature [2]. Male individuals of *H*. *zea* have three lobes at the base of the vesical, while *H*. *armigera* have one single lobe at the base of vesical [2].The female genitalia of *H*. *zea* has the appendix bursae less speculated than in the bursae of *H*. *armigera*. Moreover, the number of coils in the appendix bursae vary between the two species. *Helicoverpa zea* appendix bursae has nine to 11 dilations. *Helicoverpa armigera* appendix bursae has eight to nine dilations [2]. The characters described by Hardwick [2] to identify interspecific hybrid individuals were also considered during the genital examination, since cross-matting between *H*. *zea* and *H*. *armigera* under laboratory conditions has been reported [2, 32, 33]. All identified insects’ genitalia characters were typical of either *H*. *zea* or *H*. *armigera*. Maize ears infested with *Spodoptera frugiperda* (J.E. Smith) (Lepidoptera: Noctuidae) were not considered or reported in the present study. The percentage of *H*. zea and *H*. *armigera* were calculated considering the total of adults of *Helicoverpa* emerged. The total of *H*. *zea* and *H*. *armigera* were grouped per each location and crop season.

### Lepidoptera stock colony

During the years of 2013 to 2016, stock colonies of *H*. *zea* and *H*. *armigera* were maintained under laboratory conditions (25 ± 2°C, RH: 60 ± 10%; 14:10 [L:D]) on artificial diet [34]. Portions of cotton embedded in a solution of water and honey (10%) was provided for moths. The stock colony was reared based on established protocols [34, 35], enabling a supply of larvae for the laboratory and field experiments at the São Paulo State University, College of Agronomic Science, Department of Crop Protection, Botucatu, SP, Brazil. To maintain colony vigor, insects were collected from the field every two months, identified [2] and transferred to the colony of each species. The insects from the stock colony of *H*. *zea* and *H*. *armigera* had the species identification validated following the genital morphological characters, after it’s dead, described in the previews section.

### Intraguild competition

Assays involving larvae of *H*. *zea* and *H*. *armigera* were performed to evaluate the intraguild competition. The assays were performed under laboratory conditions at LARESPI—Laboratory of Host Plant Resistance and Insecticidal Plants (25 ± 2°C, RH: 60 ± 10%; 14:10 [L:D]) and under field conditions during two crop seasons (May to August of 2015, and November of 2015 to February of 2016) at the São Paulo State University, Botucatu, SP, Brazil (22°82’48”S and 48°42’80”W, 720 m elevation). The competition assays were conducted in three arenas (plastic cups in laboratory; plastic tubes in laboratory; and maize ears in field); two types of diet (non-Bt maize silks and non-Bt maize ear); and in 16 competition scenarios as described in Table 1. The non-Bt hybrid maize (Pioneer 30F35) was used to eliminate the possible effect of Bt proteins on larval interactions.

**Table 1:** *Helicoverpa armigera* and *Helicoverpa zea* competition scenarios at different instar.

Intraguild competition scenarios (16) ^a^	
Treatments (instar)	Controls^b^ (instar)
*H*. *armigera* (2^nd^) vs. *H*. *zea* (2^nd^)	*H*. *armigera* (2^nd^) vs. *H*. *armigera* (2^nd^)
*H*. *armigera* (2^nd^) vs. *H*. *zea* (4^th^)	*H*. *armigera* (2^nd^) vs. *H*. *armigera* (4^th^)
*H*. *armigera* (4^th^) vs. *H*. *zea* (2^nd^)	*H*. *armigera* (4^th^) vs. *H*. *armigera* (2^th^)
*H*. *armigera* (4^th^) vs. *H*. *zea* (4^th^)	*H*. *armigera* (4^th^) vs. *H*. *armigera* (4^th^)
*H*. *zea* (2^nd^) vs. *H*. *armigera* (2^nd^)	*H*. *zea* (2^nd^) vs. *H*. *zea* (2^nd^)
*H*. *zea* (2^nd^) vs. *H*. *armigera* (4^th^)	*H*. *zea* (2^nd^) vs. *H*. *zea* (4^th^)
*H*. *zea* (4^th^) vs. *H*. *armigera* (2^nd^)	*H*. *zea* (4^th^) vs. *H*. *zea* (2^nd^)
*H*. *zea* (4^th^) vs. *H*. *armigera* (4^th^)	*H*. *zea* (4^th^) vs. *H*. *zea* (4^th^)

^a^ Larval development: 4–12 h after ecdysis

^b^ Control treatments [36]

#### Laboratory intraguild competition

Two competition studies were conducted under laboratory conditions. In the first study, two larvae (Table 1) were placed into a transparent plastic cup (5 cm diameter, 100 mL) containing maize silks and enclosed with a plastic lid with holes to allow ventilation. For the second study, two larvae were placed on the silks of a maize ear, that was fixed on a base of polystyrene, supported by two wooden rods and held in place by a rubber band. The stabilized maize ear was then placed in a transparent plastic cylinder (8 cm height x 30 cm diameter), sealed on the top with organdy fabric attached with a rubber band to allow adequate ventilation. Each plastic cup or tube was considered one replicate, with 20 replicates per scenario for both arenas in a completely randomized design.

Maize silks (100 g) and maize ears were collected from non-Bt field-grown maize, which were not proximal to Bt maize fields to avoid possible cross-pollination with Bt maize [37, 38]. Maize silks were removed from the ears, sprayed with alcohol, followed by water, and then dried before being offered to larvae. Silks were changed daily to maintain quantity and tissue quality. The maize ears were cleaned with alcohol and a portion of paper towel was fixed at the base of the ear using a rubber band. The paper towel was moistened every two days to maintain ear turgidity. Ears in stage R1 were selected to obtain silks, and ears between stages R2 (blister stage) and R3 (milk stage) [29] were selected for the on-ear competition scenarios.

Larvae used in this study were removed from the artificial diet and kept individualized in plastic cups one hour to fast before each assay. For the competition study with maize silks, evaluations of larval survival were performed daily for a period of 10 days. For the competition study using maize ear, the evaluations of larval survival were performed 10 days after infestation due to the difficulty in accessing larvae in the ear every day.

Blue and red paint (Luminous Paints Kit, BioQuip Products, Rancho Dominguez, CA, USA) were utilized to identify larvae in the scenario of *H*. *armigera* and *H*. *zea* (2^nd^ vs. 2^nd^ or 4^th^ vs. 4^th^) in plastic cups. Larvae were checked twice a day and repainted after each molt. In competition study conducted on maize ears (in the laboratory and in the field), the surviving larvae were kept individualized in laboratory on the same diet. After moth emergence, species identification was carried out through examination of the morphological characteristics of genitalia [31].

#### Field intraguild competition

The field competition studies were carried out in non-Bt maize fields grown using standard agronomic practices recommended for the region of Botucatu, SP, Brazil. Natural infestations of *S*. *frugiperda*, *H*. *zea*, and *H*. *armigera* were managed using the insecticide clorfluazuron (Atabron, ISK Ltd., Indaiatuba, Brazil) during vegetative stages. During the reproductive stages, insecticide applications ceased and eggs, larvae and moths were manually eliminated when detected.

For each planting date, an area of approximately 1500 m^2^ was divided into five blocks, evenly spaced, with 10 plots each (corresponding to the competition scenarios). Four replicates were established in each plot in a randomized complete block design. Each plot was 4 m long with three rows, spaced 0.70 m apart, corresponding to approximately 22 m^2^. In each plot, only the central row was used for the competition scenario evaluations.

When the plants reached R2-R3, each maize ear was infested with larvae, placed on the region of the silks (Table 1), and the maize covered with an organdy fabric bag (25 x 30 cm) [39]. The survival of larvae was assessed 10 days after infestation.

### Statistical Analyses

The proportions of *H*. *zea* and *H*. *armigera* sampled in maize ears were calculated and expressed in percentage. Larval survival data from the different lab and field studies of larval competition were assessed for normality with Shapiro-Wilks tests. The data were tested using Chi-square test (*x*^2^) (*P ≤* 0.05) (CHISQ option, PROC FREQ, SAS Institute 2001) between the survival in the competition treatment scenario and its corresponding control [40]. The control treatment for each competition scenario consisted of individuals of the same species, in the same instars as larvae from the corresponding treatments (Table 1).

## Results

### Occurrence and larval percentage of *H*. *zea* and *H*. *armigera* in maize fields in Brazil

**Fig 1:**
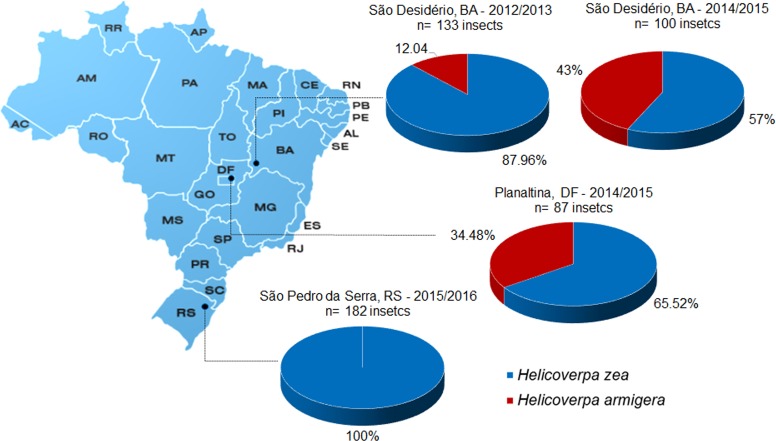
Infestation of *Helicoverpa zea* and *Helicoverpa armigera* in maize ears, in three Brazilian regions.

Overall, some larval mortality was observed during the transport of the larvae collected, in part because of parasitism by Diptera and wasps from the Ichenumonidae family [41]. In western Bahia, from the 200 larvae collected in the 2012/2013 crop season, 133 adults emerged. Examination of adult female and male genitalia indicated 117 *H*. *zea* (87.96%) and 16 *H*. *armigera* (12.04%) (Fig 1). In the 2014/2015 crop season, from 100 adults that emerged, 57 were identified as *H*. *zea* and 43 were *H*. *armigera*. In central Brazil, from 87 adults that emerged, 57 (65.52%) adults were *H*. *zea* and 30 (34.48%) were *H*. *armigera*. In the collection from southern Brazil, from 182 adults that emerged, 100% were *H*. *zea*. *Helicoverpa armigera* was not found in any collections from samples in the south. More than one larvae per ear was never observed in any collection.

### Intraguild competition

In laboratory trials with maize silks, the survival of *H*. *armigera* did not differ significantly in competitions of 2^nd^ vs. 2^nd^ instar against *H*. *zea* or *H*. *armigera*, where the survival ranged between 20 and 35% (Table 2). There were no significant differences between the survival of *H*. *armigera* on maize silks when the competitions were between 2^nd^ vs. 4^th^ instar (0% survival) and 4^th^ vs. 2^nd^ instar (100% survival). In scenario 4^th^ vs. 4^th^ instar, with maize silks, survival of *H*. *armigera* was lower in intraspecific competition (0% survival), compared to the scenario against *H*. *zea* (25% survival).

**Table 2:** Survival of *Helicoverpa armigera* in intraguild competition against *Helicoverpa zea* and in intraspecific competition by instar, in maize silks and ears, in the laboratory and field.

Site	Scenario ^a^		Survival of *H*. *armigera* (%)	Scenario		Survival of *H*. *armigera* (%)		
	*H*. *armigera*	*H*. *zea*		*H*. *armigera*	*H*. *armigera*		*x*^*2*^	*P* ^b^
Silks	2^nd^	2^nd^	35	2^nd^	2^nd^	20	1.12	0.2881
Silks	2^nd^	4^th^	0	2^nd^	4^th^	0	-	-
Silks	4^th^	2^nd^	100	4^th^	2^nd^	100	-	-
Silks	4^th^	4^th^	0	4^th^	4^th^	25	5.71	0.0168
Ear	2^nd^	2^nd^	20	2^nd^	2^nd^	30	0.53	0.4652
Ear	2^nd^	4^th^	15	2^nd^	4^th^	20	0.17	0.6673
Ear	4^th^	2^nd^	100	4^th^	2^nd^	95	1.02	0.3112
Ear	4^th^	4^th^	25	4^th^	4^th^	30	0.12	0.7233

^a^ First column, *H*. *armigera* (treatment); second column, competitor

^b^ *P* value related to the comparison the survival in intraspecific and interspecific competition.

In trials with maize ears, the survival of *H*. *armigera* did not differ among the competition scenarios, with the percentage of survival from 95% in 4^th^ vs. 2^nd^ instar scenario and between 15 and 30% in the other scenarios.

**Fig 2:**
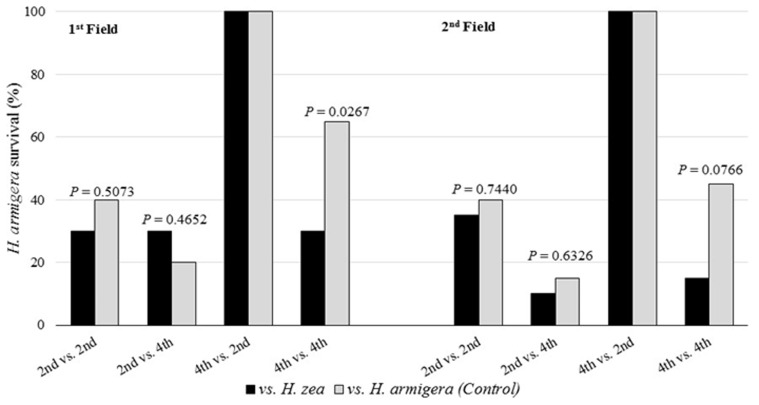
Survival of *Helicoverpa armigera* in intraspecific and interspecific interactions in field conditions in two crop seasons.

Regarding the first field competition study (Fig 2), there were no significant differences in *H*. *armigera* survival between the scenarios involving *H*. *zea* and the control, except in scenario 4^th^ vs. 4^th^ instar, where *H*. *armigera* had 65% survival in competition against its conspecific and 30% against *H*. *zea* (*x*^2^ *=* 4.91; df = 1; *P* = 0.0267). For the second field study, there were no significant differences in *H*. *zea* survival between the scenarios against *H*. *zea* or in intraspecific competition, except in scenario 4^nd^ vs. 4^th^ instar, where the survival of *H*. *armigera* was higher in intraspecific competition compared to the survival against *H*. *zea* (*x*^2^ *=* 4.29; df = 1; *P* = 0.0384), with 45 and 15% survival, respectively.

In laboratory trials with maize silks for *H*. *zea*, (Table 3), the survival of *H*. *zea* was significantly higher in competitions against *H*. *armigera* (75%) than against *H*. *zea* (0%) (*x*^2^ *=* 24.00; df = 1; *P <* 0.0001). There were no significant differences in survival of *H*. *zea* larvae in scenario 2^nd^ vs. 4^th^ instar and 4^th^ vs. 2^nd^ instar, with the larvae having 0% survival in the first case and 100% survival when larvae were larger than the competitor. In the scenario 4^th^ vs. 4^th^ instar, larvae of *H*. *zea* continued to show greater survival in competition against *H*. *armigera* (75%) than in competition against *H*. *zea* (0%) (*x*^2^ *=* 24.00; df = 1; *P <* 0.0001), similar to that observed in scenario 2^nd^ vs. 2^nd^.

**Table 3:** Survival of *Helicoverpa zea* in intraguild competition against *Helicoverpa armigera* in intraspecific competition by instar, in maize silks and ears, in the laboratory and field.

Site	Scenario ^a^		Survival of *H*. *zea* (%)	Scenario		Survival of *H*. *zea* (%)		
	*H*. *zea*	*H*. *zea*		*H*. *zea*	*H*. *armigera*		*x*^*2*^	*P* ^b^
Silks	2^nd^	2^nd^	0	2^nd^	2^nd^	75	24.00	<0.0001
Silks	2^nd^	4^th^	0	2^nd^	4^th^	0		-
Silks	4^th^	2^nd^	100	4^th^	2^nd^	100	-	-
Silks	4^th^	4^th^	0	4^th^	4^th^	75	24.00	<0.0001
Ear	2^nd^	2^nd^	30	2^nd^	2^nd^	80	10.10	0.0015
Ear	2^nd^	4^th^	10	2^nd^	4^th^	30	2.50	0.1138
Ear	4^th^	2^nd^	95	4^th^	2^nd^	100	1.02	0.3112
Ear	4^th^	4^th^	10	4^th^	4^th^	70	15.00	0.0001

^a^ First column, *H*. *zea* (treatment); second column, competitor

^b^ *P* value related to the comparison the survival in intraspecific and interspecific competition.

On maize ears, the results of *H*. *zea* survival were similar to those observed on maize silks (Table 3). There was no difference in the scenario 2^nd^ vs. 4^th^ instar and 4^th^ vs. 2^nd^ instar. In the scenario of 2^nd^ vs. 2^nd^ instar, *H*. *zea* larvae had 80% survival in competition against *H*. *armigera* (*x*^2^ *=* 10.10; df = 1; *P =* 0.0015), while in intraspecific competition, the survival was 30%. When competing in scenario 4^th^ vs. 4^th^ instar, the survival of *H*. *zea* remained high against *H*. *armigera* (70%), and low in intraspecific competition (10%) (*x*^2^ *=* 15.00; df = 1; *P =* 0.0001).

For both field competition studies, the results were similar for the scenarios of 2^nd^ vs. 2^nd^ instar and 4^th^ vs. 4^th^ instar (Fig 2). In the first scenario, *H*. *zea* survival was above 75% in competition against *H*. *armigera*, while in intraspecific competition the survival was below 10% (*x*^2^ *=* 19.79; 20.42; df = 1; *P* < 0.0001). In scenario 4^th^ vs. 4^th^ instar, survival of *H*. *zea* remained higher in competition against *H*. *armigera* (above 65%) in relation to survival against *H*. *zea* (0%) (*x*^2^ *=* 26.66; 19.26; df = 2; *P <* 0.0001). In the first field competition study, there were no significant differences in the scenario of 2^nd^ vs. 4^th^ instar and 4^th^ vs. 2^nd^ instar. In the second study, there was a significant difference in the scenario of 2^nd^ vs. 4^th^ instar, when the survival was higher in competition against *H*. *armigera* (35%) compared to the control (*x*^2^ *=* 8.48; df = 1; *P =* 0.0036). No difference was observed for the scenario 4^th^ vs. 2^nd^ instar in the second competition study.

## Discussion

Larval survival in plastic cups with maize silks was lower than larval survival in ears (Tables 2 and 3). Although larval survival was slightly lower in the laboratory than in the field, in general the behaviors and relationships observed under both sites were similar. The field study used arenas that allowed the larvae in maize ears to be isolated from the external environment and natural predators, parasitoids or other food competitors, which can be significant causes of larval mortality [42]. This demonstrates the importance of the competition arena size and the food source. The higher survival in maize ears might be related to a greater quantity and higher quality of food for the larvae [43] compared to simply silks, which provide less nutritional value [44] than maize ears (i.e. silks and kernels). In addition, larval encounters in the maize ear may be less frequent due to the larger area available for escape, and therefore less chance of an aggressive encounter, which is a larval response during larval interaction [45, 46]. Overall, results indicate that the maize ear is an appropriate arena to study intraguild competition between *H*. *zea* and *H*. *armigera*, and among other ear feeding noctuid species.

**Fig 3:**
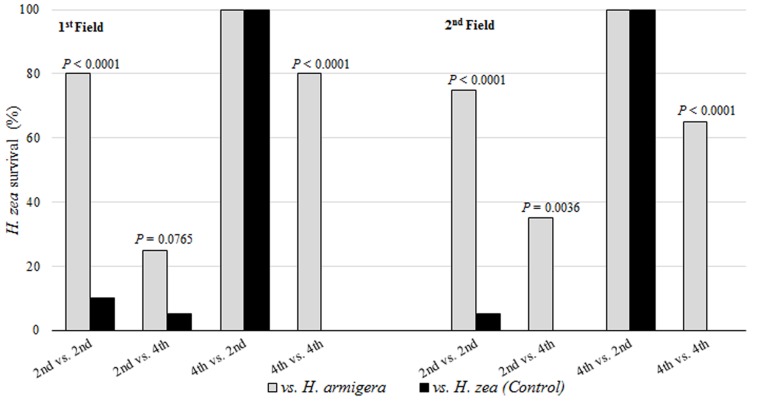
Survival of *Helicoverpa zea* in intraspecific and interspecific interactions in field conditions in two crop seasons.

*Helicoverpa zea* exhibited the highest level of cannibalism, mainly in plastic cups with maize silks (limited food and small arenas). Cannibalism is dependent on instar, with higher rates in later instars, and especially when larvae of different instars interact [47–49]. Cannibalism in *H*. *zea* may occur at rates of up to 75% [48, 50]. Cannibalism and intraguild predation are treated as direct benefits when food is limited, where one larva preying on others can result in fitness benefits and increase the rate of development, body mass and fecundity [12, 48, 49], or as an indirect benefit by the exclusion of a potential food competitor [47, 48]. Negative effects of cannibalism may be injury and subsequent death, or acquisition of pathogens or parasites and consequent reduction in larval fitness and development [49–54]. These results suggest the aggressive behavior of *H*. *zea* is higher than that of *H*. *armigera* in intraspecific competition scenarios (Tables 2 and 3) (Figs 2 and 3). The impact of aggressiveness and the cannibalism in *H*. *zea* populations help to explain why the occurrence of more than one larvae in a maize ear is rare [55], and was not observed during larval collections in maize in different regions in Brazil.

The introduction of *H*. *armigera* in Brazil and other countries in the American continents, presents the opportunity and need to investigate the interaction of these former allopatric species. An initial result is the possible occurrence of hybrids between the two species, which until now has only been documented in the laboratory under artificial conditions [2, 56–58]. An examination of all specimens collected from the field did not reveal any variability in female and male genital characters as described by Hardwick (1995) that would indicate the occurrence of hybrids from cross-mating between *H*. *zea* and *H*. *armigera* under natural conditions.

Another aspect of *H*. *armigera’s* introduction is the possible interspecific competition between these two species. Previously, *H*. *zea* and *H*. *armigera* were considered one cosmopolitan species, referred to as either *Heliothis obsolete* or *H*. *armigera* [2]. Based on taxonomic investigations in different regions of the world, an actual complex of species was suggested under the new genus *Helicoverpa*, the *armigera* group represented by two species in the Old World and the *zea* group represented by eight species distributed in America continents [2]. This increasing acknowledgment of Heliothinae systematics [59, 60] indicated *H*. *zea* and *H*. *armigera* originated from a common ancestor and were geographically isolated and defined as allopatric species [2, 58, 59].

The maize ear is an appropriate arena for intraguild interaction studies. Natural infestation of both species occur during the reproductive stage [14, 19], and exhibit similar development [61, 62], which increases the possibility of interaction. Although a challenge in the competition studies was the precise identification of the species of the insect that survived, differentiation between *H*. *zea* and *H*. *armigera* is possible with the dissection and examination of genitalia characteristics [31].

The preliminary expectation of the interaction between *H*. *zea* and *H*. *armigera* was that *H*. *armigera* would be favored, since it was recently detected as an invasive species in Brazil [4–6] it would have all advantages that a new organism typically has in a new environment [63]. However, the intermediate to low survival of *H*. *armigera* in competition scenarios 2^nd^ (vs. 2^nd^), 2^nd^ (vs. 4^th^) and 4^th^ (vs. 4^th^) (Table 2) (Fig 2) suggests that this species is negatively affected in intraspecific competition (with a considerable percentage of cannibalism), and in interspecific competition (intraguild predation) on non-Bt maize. In competition scenarios 2^nd^ (vs. 2^nd^) and 4^th^ (vs. 4^th^), the low survival of *H*. *armigera* in intraspecific competition and against *H*. *zea* indicates the presence of cannibalism and the disadvantage of larvae competing with *H*. *zea* of the same instar (Table 2) (Fig 2). The results of 4^th^ vs. (4^th^) in the field support this disadvantage, where 80% survival of *H*. *zea* was observed (Fig 3). The low survival of *H*. *amigera* in 2^nd^ (vs. 4^th^) competitions, with zero survival on maize silks, suggests the importance of the arena size and food source during an intraguild competition. This scenario also indicates the importance of larval stadia during a competition, where the smaller insect has considerable disadvantages. Similarly, in 4^th^ vs. (2^nd^) competition, the high larval survival indicates the importance of the instar in intraguild competition between the species, and only in this case does larvae of *H*. *armigera* have a high probability of gaining advantage over *H*. *zea*. Some studies have reported similar development between these species [10, 61, 62, 64], however, a recent study conducted on artificial diet indicated that *H*. *zea* are larger than *H*. *armigera*, and have slower development [64]. Although the study was conducted on artificial diet, it indicates that biological differences between the species may occur, and along with host plant and other factors, influence intraguild competition.

Overall, in the interspecific competition, *H*. *zea* larval survival was higher when in competition against *H*. *armigera*, even when *H*. *zea* was smaller than *H*. *armigera*. The survival of *H*. *zea* indicates the potential of this species to gain advantage in intraguild competition, as was observed in the second crop season.

The percentage of *H*. *zea*:*H*. *armigera* in maize ears determined in natural infestations indicates the prevalence of *H*. *zea*, and supports the results observed in the competition studies. However, it is important to highlight that the prevalence of *H*. *zea* was variable in different regions. In Rio Grande do Sul, *H*. *armigera* was not detected in the maize ears (Fig 1), even though this species was reported as present in southern Brazil from at least 2011 [7]. In Planaltina, DF (central Brazil), the proportion of *H*. *zea*: *H*. *armigera* larvae in maize ears was approximately 2:1 (Fig 1). In western BA, two different infestation events and proportions of *H*. *zea*:*H*. *armigera* larvae in maize ears were documented. During the 2012/2013 crop season, when the first outbreak of the *H*. *armigera* in the region was reported [4–6], the proportion of *H*. *zea*: *H*. *armigera* larvae in maize ears was 7:1. In the following crop season (2013/2014), an increase of the proportion of *H*. *armigera* to 1:1 larvae in maize ears was observed (Fig 1).

These field data from natural infestations in maize indicate that besides the aggressive behavior of *H*. *zea*, the region-specific landscape, and especially the presence of cotton, may play a role in the presence and proportion of *H*. *armigera* in maize. Cotton is a preferred host of *H*. *armigera* [65–67]. In Rio Grande do Sul there is no cultivation of cotton. In Planaltina, DF (central Brazil), cotton is present, but not a prevalent crop in a diversified mosaic of several hosts, including soybean, maize, dry beans, and vegetables, among others. In western BA, the landscape is a mosaic formed by an ocean of soybean and patches of maize and cotton. It is possible that because of the higher proportion of cotton than maize in the western BA landscape, *H*. *armigera* has been able to successfully establish and build high populations. These populations feed in maize as an alternative host, especially in the beginning of the crop season because maize is the first crop planted in the region. Moreover, these results indicate a significant presence of *H*. *armigera* in maize even when in competition with the aggressive *H*. *zea*. This hypothesis is supported by the absence of *H*. *armigera* in maize in a state that does not cultivate cotton, and the increase of the proportion of *H*. *armigera* in maize in western BA from the 2012/2013 to the 2014/2015 crop seasons.

In an IPM perspective, these results indicate that maize and cotton could play a role as a reservoir of *H*. *armigera* in the landscape. *Helicoverpa armigera* has preference for reproductive plant stages and maize has a relatively short flowering stage. In consequence, maize supports only one generation of this species [19]. However, the cultivation of maize all year round in Brazil provides the opportunity for maize to be a source of infestation of *H*. *armigera*, even though competition with *H*. *zea* will in part regulate the population density of *H*. *armigera* on non-Bt hybrids.

On the other hand, these results should be also be considered with respect to insect resistance management to insecticides and to Bt technology. Maize may be considered a source of *H*. *armigera* unselected by insecticides commonly used in cotton, such as emamectin benzoate [68, 69]. Non-Bt maize may also support populations of *H*. *armigera* that are not exposed to Bt proteins. This is the case for Cry1Ac and Cry2Ae expressed in some Bt cotton events [70–72], and Cry1Ac in Bt soybean, which is cultivated in large areas in Brazil since the 2014/2015 crop season.

In the savanna areas of Brazil, with a prevalence of maize, soybean and cotton, it is important to investigate how the agroecosystem will define the prevalence and the impact of these two former allopatric species. In the same way, because of the recent detection of *H*. *armigera* in the United States [10], it is important to consider the variable agroecosystems of North America (e.g. Corn Belt, Cotton Belt) on how *H*. *zea* and *H*. *armigera* will ultimately impact IPM and IRM in the Americas.
